# Mite communities (Acari: Mesostigmata) in young and mature coniferous forests after surface wildfire

**DOI:** 10.1007/s10493-017-0148-4

**Published:** 2017-06-20

**Authors:** Jacek Kamczyc, Cezary Urbanowski, Emilia Pers-Kamczyc

**Affiliations:** 1 Department of Game Management and Forest Protection, Poznań University of Life Sciences, Wojska Polskiego 71C, 60-625 Poznań, Poland; 2 Institute of Dendrology, Polish Academy of Science, Parkowa 5, Kórnik, Poland

**Keywords:** Wildfire, Forest age, Scots pine, Mites, Mesostigmata

## Abstract

Density, diversity and assemblage structure of Mesostigmata (cohorts Gamasina and Uropodina) were investigated in Scots pine forests differing in forest age (young: 9–40 years and mature: 83–101 years) in which wildfire occurred. This animal group belongs to the dominant acarine predators playing a crucial role in soil food webs and being important as biological control agents. In total, six forests (three within young and three within mature stands) were inspected in Puszcza Knyszyńska Forest Complex in May 2015. At each forest area, sampling was done from burned and adjacent control sites with steel cylinders for heat extraction of soil fauna. Data were analyzed statistically with nested ANOVA. We found a significant effect on mite density of both fire and forest age, with more mites in mature forests and control plots. In total, 36 mite taxa were identified. Mite diversity differed significantly between forest ages but not between burned versus control. Our study indicated that all studied forests are characterized by unique mite species and that the mite communities are dominated by different mite species depending on age forest and surface wildfire occurrence. Finally, canonical correspondence analysis ranked the mite assemblages from control mature, through burned young and burned mature, away from the control young.

**Electronic supplementary material:**

The online version of this article (doi:10.1007/s10493-017-0148-4) contains supplementary material, which is available to authorized users.

## Introduction

Fire is a dominant large-scale disturbance factor in many of the world’s terrestrial ecosystems including forests (Malmström 2012). It can affect both the above ground part of the forest ecosystems by burning the shrubs and trees and also the soil environment (Buhk et al. 2007; Certini 2005). The impact of fire on soil depends on many factors such as forest characteristics (amount, nature and moisture of life and dead fuel), climatic conditions (air temperature and humidity, wind spread), topography of the site and also fire type and severity (intensity and duration) (Lóšková et al. 2013). Recent studies have indicated that the climatic changes (i.e. rising temperatures and water stress) are expected to have a great impact on fire risk around the globe (Moriondo et al. 2006). This assumption applies to temperate forests in Southern and Central Europe (Allen et al. 2010) as climatic changes will lead to a more pronounced continental climate characterised by a higher occurrence of droughts and fire danger (Gerstengabe et al. 1999). Currently, forests in Europe are annually influenced by hundreds of thousands of fires which cover hundreds hectares of forests area (ECJRC 2016). Although the average area burned per fire is rather low, due to the availability and efficiency of fire-fighting resources and infrastructures (Gerth 2001), recent reports of the European Commission Joint Research Centre on Forests (ECJRC 2016) concluded that the area burned by forest fires in the European Union could double by the end of the century as a consequence of climate change.

Temperate forests in Europe are mostly formed by Scots pine (*Pinus sylvestris* L.) trees. This tree species has an immense distribution that extends the breadth and width of Europe and Asia (Rehfeldt et al. 2002; Bernhardsson et al. 2016), has a broad ecological tolerance and is growing on a wide range of soils under varying climatic regimes (Bradshaw and Browne 1987). In general, Scots pine is growing in cultivations characterized by similar age. Young and mature forests have different characteristics, such as the amount of combustible plant material (e.g. tree density, number of dead and decaying trees, litter input). Those differences can determine the risk of transformation of a fire from surface fire into crown fire due to the high stem density and high ladder fuel connectivity between the ground and canopy in young forests (Kobziar Leda et al. 2009).

Fire can indirectly affect the soil animal communities by changes in habitat conditions and removal of food sources reflected by organic matter, water-holding capacity and structural complexity of soil. However, it can also have a direct effect on the mortality of soil animals due to heat exposure (Camann et al. 2012). Previously published studies connecting fire and soil animal communities (Table 1) have focused on two aspects: the recovery process and the effect of various types of fires on soil fauna communities. The recovery process after the fire was investigated in short (until 1 year) (Badejo 1994; Camann et al. 2007) versus long (for years) periods of time (Kudryasheva and Laskova 2002; Bogorodskaya et al. 2010; Kim and Jung 2013), both after wildfire (Hylander 2011; Kim and Jung 2013; Lóšková et al. 2013; Zaitsev et al. 2014) or experimental burning (Bogorodskaya et al. 2010; Camann et al. 2012; Malmström 2012). There is also some research focusing on biodiversity as the effect of various types of fire (Michalik et al. 2004; Jung et al. 2010; Zaitsev et al. 2014). The latest research has pointed towards changes within abundance and species richness in relation with fire severity (Kim and Jung 2008; Jung et al. 2010) and differences in species richness and soil fauna abundances between forests in different age classes (Johansson et al. 2016). However, there is still lack of information about the relation of forests age classes and surface wildfires, which are very common in Central Europe. Nevertheless, the published studies that have been conducted in young (Jung et al. 2010; Kim and Jung 2013) and mature forests (Kudryasheva and Laskova 2002; Camann et al. 2007; Malmström 2008; Hylander 2011; Camann et al. 2012; Malmström 2012; Lóšková et al. 2013; Zaitsev et al. 2014) did not show any pattern of the relationship between forest age and fire. This may result from studies done on different tree species (e.g. Huebner et al. 2012; Camann et al. 2007) or even types of forest ecosystems from rainforests (Badejo 1994), through boreal forests (Hylander 2011) to temperate forests (Jacobs et al. 2015).

**Table 1:** Soil fauna studies in burned forests

Forest description, location and type of fire	Sampling (time of burning/fire, time of sampling)	Animal group	Citation
*Europe*			
**TF:** Scots pine forest, renewal of stand at Spring 1994	**FO:** 10 August 1992	Gamasina	Michalik et al. (2004)
**A:** middle aged (60 years-old)	**TS:** Spring 1994—after the renewal of stand		
**L:** Puszcza Notecka Forest, Poland			
**F:** wildfire			
**TF:** Scots pine forest, clear-cut in 2002	**FO:** 12 May 2004	Collembola, Oribatida, Mesostigmata, Protura	Malmström (2008)
**A:** mature (107 year-old)	**TS:** 2–3 years after fire		
**L:** Tierp, 50 km north of Uppsala, eastern central Sweden			
**F:** laboratory experimental burning			
**TF:** Fennoscandian boreal forest, pine/spruce forest with a component of aspen	**FO:** 7 years and 2–3 years before collecting	Gastropoda	Hylander (2011)
**A:** mature	**TS:** 9 August–19 September 2006, 26 June–4 September 2007		
**L:** southern Stockholm county, south-western Västernorrland, Sweden			
**F:** wildfire, prescribed burning			
**TF:** Scots pine forest **A:** mature (115-year-old) **L:** Bjuråker, central Sweden **F:** experimental fire, clear-cut burning	**FO:** 3 June 1999 **TS:** during 10 years (starting in November 1999, November/December in 1999–2001 and 2005–2008, April in 2003 and 2004)	Collembola	Malmström (2012)
**TF:** spruce forest (wind throw in 2004) **A:** mature (120 year-old) **L:** High Tatra National Park, Slovakia **F:** wildfire after windstorm	**FO:** 2005 **TS:** April and September 2007	Oribatida	Lóšková et al. (2013)
**TF:** Scots pine forest	**FO:** August 2001	Nematoda	Zaitsev et al. (2014)
**A:** mature (150–300 year-old)	**TS:** November 2008	Collembola	
**L:** 20 km south of Stockholm, central Sweden		Oribatida Mesostigmata Enchytraeidae	
**F:** wildfire			
*North America*			
**TF:** ponderosa pine forest	**FO:** 1997	Mesostigmata, Prostigmata, Oribatida	Camann et al. (2007)
**A:** mature (no age data)	**TS:** June, August and October 1998		
**L:** Southern Cascade Range of California, USA			
**F:** low intensity prescribed fire, clear-cut and burning			
**TF:** ponderosa pine forest	**FO:** October 1997	Mesostigmata, Prostigmata, Oribatida	Camann et al. (2012)
**A:** mature (no age data)	**TS:** 3 times (June 1998, October 1998, June 1999)		
**L:** Southern Cascade Range of California, USA			
**F:** low intensity prescribed fire			
**TF:** maple-oak forest	**FO:** April 2009, 1960 (50 years before collecting) and >100 years before collecting	Collembola	Huebner et al. (2012)
**A:** no data	**TS:** three months after April fire (October 2009)		
**L:** Gault Nature Reserve, Québec, Canada			
**F:** ground fire			
**TF:** temperate forest, oak woodland	**FO:** periodic burn (every 3–4 years, 1986–2010), annual burn (1986–2010)	Invertebrata	Jacobs et al. (2015)
**A:** no data	**TS:** 3 times (October 2008, May 2009, October 2009)		
**L:** DuPage County, Illinois, USA, Arboretum’s East Woods			
**F:** low intensity prescribed burning			
*Asia*			
**TF:** 1. Pine and spruce boreal forest, Bilberry-moss spruce stand, 2. Shrub-peat moss pine stand	**FO:** 1 July 1972	Oribatida	Kudryasheva and Laskova (2002)
**A:** mature (60–160 years old)	**TS:** for 5 years (1973–1977), except the second year (1974)		
**L:** Onega Region, Arkhangelsk District, Russia			
**F:** moderate-rate ground fire			
**TF:** Japanese pine forest	**FO**: April 2000	Mesostigmata	Jung et al. (2010)
**A:** middle aged (≤40 years-old stand)	**TS**: 1 year after burning (2001)		
**L:** Samcheok, Gangwon province, Korea			
**F:** during east coast mountain wildfire			
**TF:** dwarf-shrub-lichen-green-moss pine forest	**FO:** 2000–2003	Collembola, Oribatida, Mesostigmata	Bogorodskaya et al. (2010)
**A:** uneven-aged	**TS:** 1 day after fire, annually during 5 years		
**L:** Sym Plain, West Siberian Plain, Russia			
**F:** experimental ground fire			
**TT:** Japanese pine forest	**FO:** April 2000	Oribatida	Kim and Jung (2013)
**A:** young (30-year-old)	**TS:** 5, 6, 7 years after burning (2005, 2006, 2007)		
**L:** Imwon-ri, Yang-ri, Samcheok, Gangwon province, Korea			
**F:** during east coast mountain wildfire			
*Africa*			
**TF:** secondary regrowth lowland rainforest	**FO**: 20 February 1993	Cryptostigmata, Mesostigmata, Prostigmata	Badejo (1994)
**A:** no data	**TS:** 1, 3, 6 months after burning		
**L:** 200 km north-east of Lagos, Nigeria, reserve			
**F:** unknown source, ground fire, herbal layer			

Symbols are as follows: *A* age of the forest, *F* type of fire, *FO* fire occurrence, *L* location, *TF* forest type, *TS* time of sampling

Recent studies on rove beetles (Johansson et al. 2016) suggest that older forests are characterized by a higher abundance and species richness; however, no studies have assessed the reaction of soil fauna to surface wildfire in young and mature Scots pine forests. One soil fauna group is free-living soil mites (Acari, Mesostigmata). Mesostigmata are important regulators of decomposition processes in forest soil ecosystems and they also occupy a high trophic level in the soil decomposition food web (Schneider and Maraun 2009). Many mesostigmatid mite species are predators on: nematodes, other mite groups, collembolans and also enchytraeids as well as small insect larvae (Karg 1993). Therefore, the presence/absence of those mites can reflect the microflora (fungi and bacteria), microfauna (nematodes), mesofauna (other mites and collembolans) and physicochemical conditions of soil such as organic matter (Jung et al. 2010).

The objective of our research was to study the effect of surface wildfire on mesostigmatid mites communities in young and mature Scots pine forests. We addressed the following hypotheses: (1) abundance and species richness of mites is reduced by surface wildfire regardless forest class age, and (2) surface wildfire reduces the population densities of large and mobile predators living in the upper layers of the litter.

## Materials and methods

### Study sites and sampling

The study was conducted in the complex of the Puszcza Knyszyńska Forest (PKF), which is located close to the state border with Belarus (North-east Poland). This forest is situated in the coldest regions of Poland (Chrzanowski 1991) and its climate has continental character with a high difference between the mean temperature of the coldest and warmest month which reaches 22 °C (Sasinowski 1995). The mean annual precipitation oscillates around 610 mm, snow covers the ground for 85–90 days and its maximum thickness fluctuates from 8 to 80 cm. The growing season in the Puszcza Knyszyńska Forest is short, begins in the first half of April and lasts about 200 days (Sasinowski 1995). The soils of the Puszcza Knyszyńska Forest are generally rather poor. Large areas are covered with loose, slightly clayey podzols formed in sand (Czerwiński 1995). Forests, which cover 70% of the total area, are dominated by Scots pine (*Pinus sylvestris*) and Norway spruce (*Picea abies*).

**Fig. 1:**
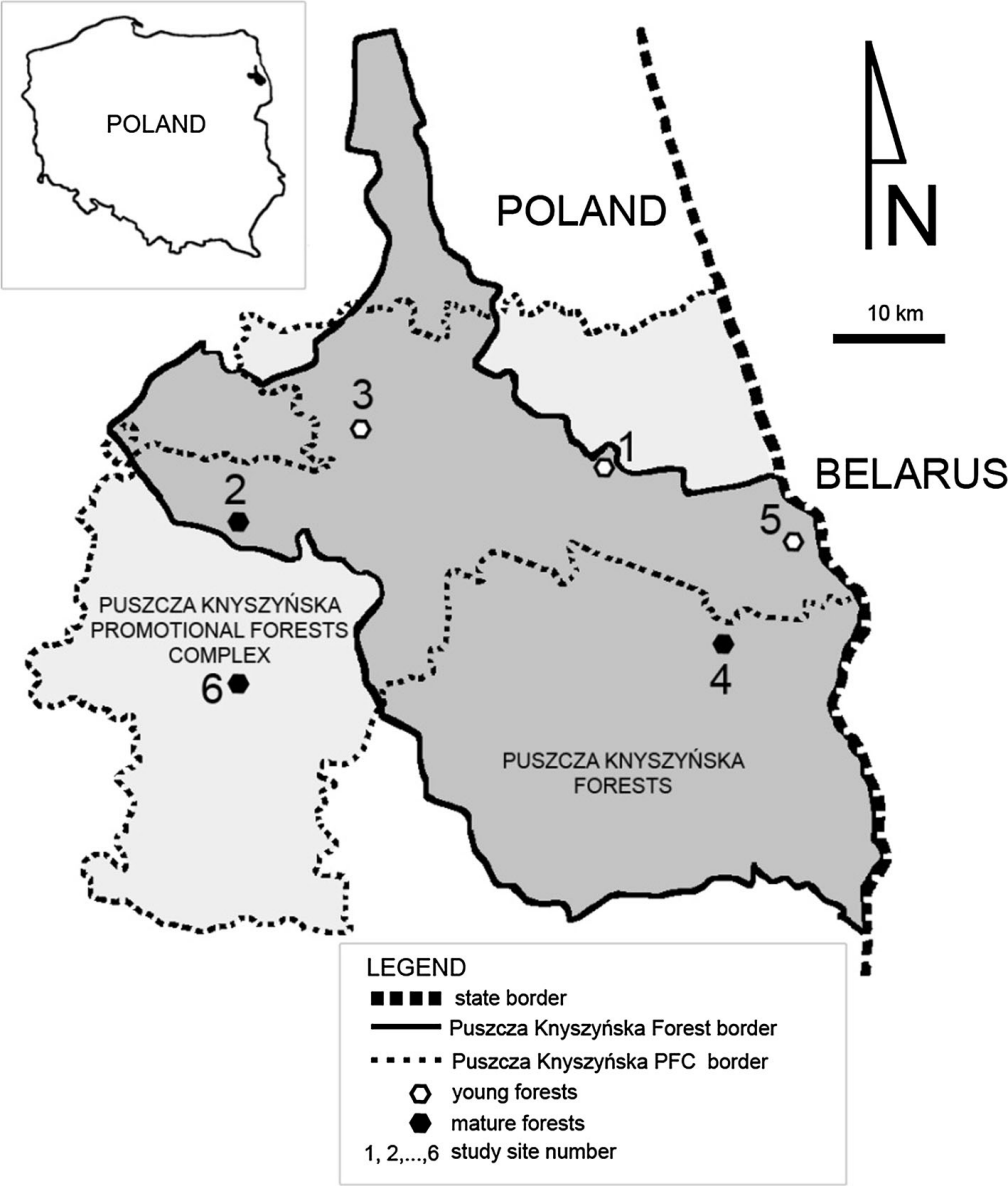
Location of the study sites on the territory of Puszcza Knyszyńska Forest

In total six study sites were selected on the territory of the PKF (Fig. 1). One site (no. 6) was located on the protected areas of the Puszcza Knyszyńska Promotional Forest Complex (PFC) as no other burned forests in the PFK suited to the studied forests. The total area of the study sites varies between 5.44 and 24.58 ha. All study sites were classified as fresh mixed coniferous forests growing on rusty soil. The understory species were represented by several species e.g. *Sorbus aucuparia*, *Picea abies*, *Quercus robur* and *Betula pendula*, and the forest floor was covered by mosses and blueberries (Table 2). On each study site the fire impact was classified as light (C2) after Jung et al. (2010) described as surface fire with high recovery and light ecosystem impact. The litter layer was burned up to the depth of ca. 3 cm and the trunks of the standing trees were affected up to the height of ca. 0.7 m. The sampled forests were divided into two groups (each group was represented by three forests): young (9–40 years) and mature (83–101 years). In each forest, two subplots (burning and a control area) were selected. Overall, 60 samples (2 groups of forest age × 3 replication × 2 subplots [burning and control] × 5 samples from each plot) were randomly collected in using steel core (40 cm^2^) to the depth of 10 cm, placed in plastic bags and stored in a portable cooler for transport to the laboratory (Poznań University of Life Sciences, Poland). The sampling was conducted in late spring (May 2015) to coincide with high invertebrate abundance.

**Table 2:** Characteristics of the study sites

Study site	Coordinates	Time of fire	Total area (ha)	Fire area (ha)	Forest stand	Age (years)	Forest type	Forest floor	Understory species	Type of soil
1	23°35′25″E 53°16′56″N	2014	5.88	0.01	Young forest	40	Fresh mixed coniferous forest	Mosses	*Sorbus aucuparia*, *Picea abies*, *Quercus robur*, *Betula pendula*, *Populus tremula*	Rusty soil
2	23°06′11″E 53°13′41″N	2014	7.35	0.15	Mature forest	83	Fresh mixed coniferous forest	Mosses, blueberries	*Sorbus aucuparia*, *Picea abies*, *Quercus robur*, *Frangula alnus*, *Corylus avellana*	Rusty soil
3	23°15′44″E 53°18′20″N	2013	5.45	0.41	Young forest	9	Fresh mixed coniferous forest	Mosses, blueberries	*Picea abies*, *Acer platanoides*, *Corylus avellana*	Rusty soil
4	23°45′60″E 53°07′28″N	2013	5.44	0.30	Mature forest	101	Fresh mixed coniferous forest	Mosses, blueberries	*Picea abies*, *Frangula alnus*, *Betula pendula*, *Juniperus communis*	Rusty soil
5	23°50′06″E 53°12′35″N	2012	24.58	0.80	Young forest	14	Fresh mixed coniferous forest	Mosses, blueberries	*Sorbus aucuparia*, *Sambucus nigra*, *Crataegus monogyna*	Rusty soil
6	23°06′45″E 53°05′56″N	2012	6.77	0.06	Mature forest	86	Fresh mixed coniferous forest	Mosses, blueberries	*Sorbus aucuparia*, *Quercus robur*, *Frangula alnus*, *Juniperus communis*, *Populus tremula*	Rusty soil

### Mite extraction and identification

Mites were extracted from samples using Tullgren type funnels (20 cm diameter) with a mesh size of approx. 2 mm. Tullgren extraction is recommended for species inventory in highly organic soils such as those in the Scots pine forest floors in this study (Crossley and Blair 1991; Edwards 1991). The extraction efficiency of this method reaches over 80% (van Straalen and Rijninks 1982). The temperature and moisture gradient in the Tullgren funnels forced active soil fauna to move down the core into 70% ethanol over a period of 7 days. Mesostigmatid mites were separated from the samples and sorted under a stereomicroscope at 10–25 × magnification, cleared in 85% lactic acid for a minimum of 3 days, depending on the degree of transparency required for each specimen, slide-mounted using Hoyer’s medium and finally dried at 45 °C for minimum 7 days using a slide warmer. The total number of mesostigmatid mites was determined using a microscope. The mites were determined by species (adults and juvenile when possible) or genus using a stereomicroscope, with keys (Micherdziński 1969; Giljarov and Bregetova 1977; Karg 1993).

### Data analysis

Each soil/litter core provided an independent estimate of local diversity and abundance. To avoid pseudoreplications, five sampling points sampled within each group obtained from study site were used to determine average mean. Abundance data were transformed into square meter scale (m^−2^) per plot for easy comparison with published data. The normality of data distribution was tested using the Shapiro–Wilk W Test. Data describing mite abundance were log-transformed to reduce skewness. ANOVA was conducted with group (control, burned) nested within forest age (young, mature). Tukey’s HSD was employed to compare differences between means. Results were considered significant when *P* < 0.05. Statistics were performed with the software package JPM (SAS Institute).

Diversity for each sample was measured using the Shannon’s diversity index (*H*′) and Eveness index (*E* = *H*′/ln[Richness]). The Shannon index was calculated using the formula *H′* = –Σ*p*_*i*_ ln[*p*_*i*_], where *H′* is Shannon’s index and *p*_*i*_ is the proportion of individuals found in the *i*-th species. Species richness was examined by counting the species in each sample. The species rank graph was restricted to the most dominant species (Dominance, *D* ≥ 0.03%). Data of density and diversity were calculated per square meter. To determine the gradient of faunistic variation we used detrended correspondence analysis (DCA), down-weighting of rare species using MVSP 3.0. The DCA was carried out for four microhabitats (two control and two burned forests) and 12 mite species. Each species was represented by at least 10 individuals.

## Results

Our study revealed the impact of the fire and the forests age on mite abundance. Higher mite abundances were observed in control forests when compared to burned plots both in young (2167 vs. 1383 individuals; *t* = 3.14, *df* = 1, *P* = 0.014) and mature forests (3817 vs. 2150 ind.; *t* = 4.09, *df* = 1, *P* = 0.0035). Moreover, mite abundance was significantly higher in the control mature forest compared to other groups and decreased as follows: young control, mature burned and finally young burned (*Q* = 3.202, *P* = 0.003).

**Fig. 2:**
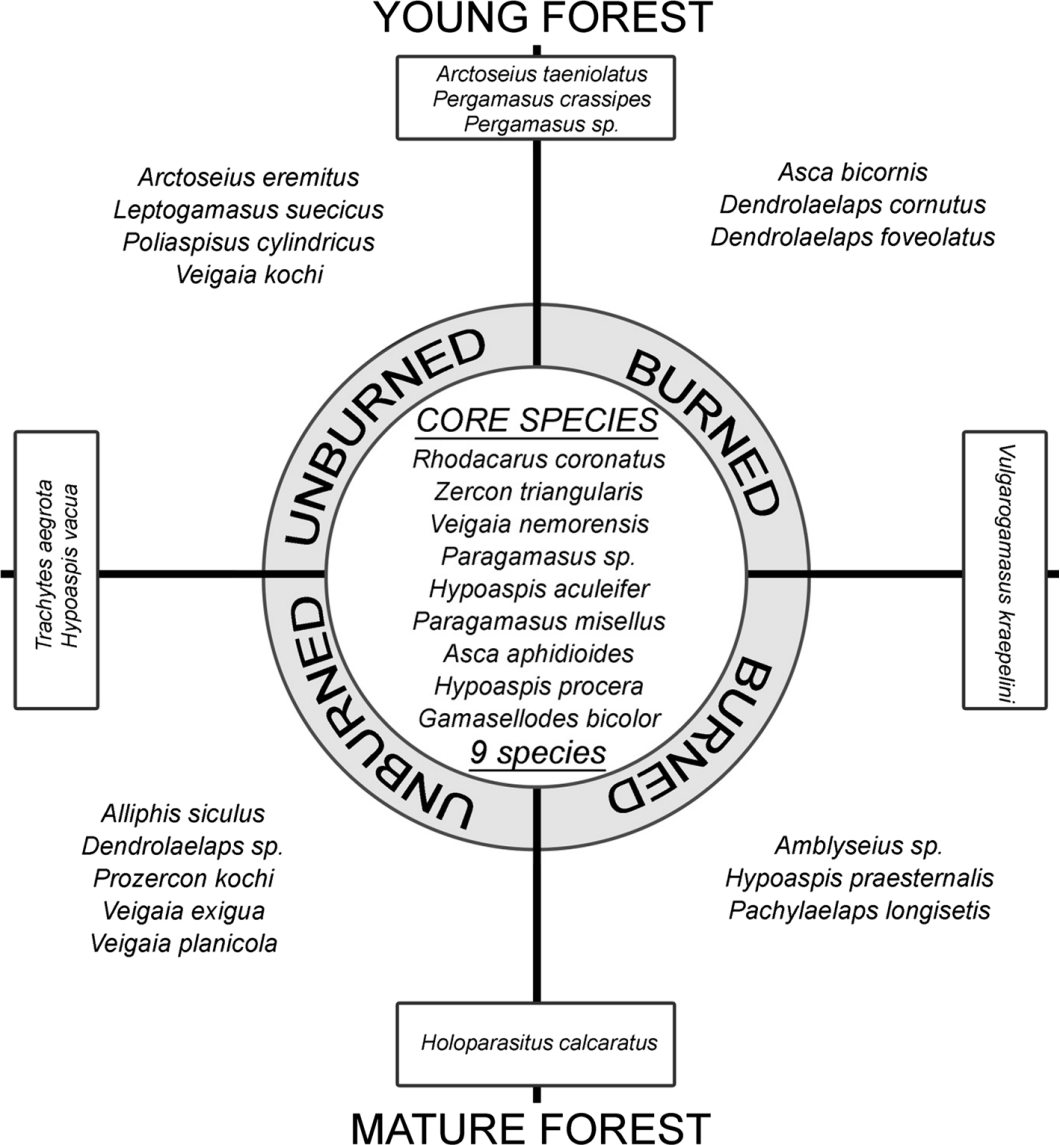
Core, exclusive and common mite species in young and mature, burned and unburned coniferous forests

In total 571 mites were recorded and classified into 36 species. Our study indicated that nine species occurred in all microhabitats (control and experimental as well as in young and mature) and play a role of core species. The core species group was represented by *Rhodacarus coronatus*, *Zercon triangularis*, *Veigaia nemorensis*, *Paragamasus* sp., *Hypoaspis aculeifer*, *Paragamasus misellus*, *Asca aphidioides*, *Hypoaspis procera* and *Gamasellodes bicolor*. Moreover, this study indicated that some species are characteristic for burned and unburned sites, both in young and mature forests, and that some species occur in two types of forests (Fig. 2). Our study revealed differences in mite communities between unburned and burned plots. The unburned forests were characterized by 11 exclusive species. They were represented by two common species regardless of a forests age (*Trachytes aegrota* and *Hypoaspis vacua)* and four species specific for young (*Arctoseius eremitus*, *Leptogamasus suecicus*, *Polyaspinus cylindricus*, *Veigaia kochi*) and five species for mature forests (*Alliphis siculus*, *Dendrolaelaps* sp., *Prozercon kochi*, *Veigaia exigua*, *V. planicola*). The burned forests were characterized by seven exclusive species. They were represented by common species regardless of a forests age (*Vulgarogamasus kraepelini*) and three species in each, young (*Asca bicornis*, *Dendrolaelaps cornutus*, *D. foveolatus*) and mature forest (*Amblyseius* sp., *Hypoaspis praesternalis*, *Pachylaelaps longisetis*) (Fig. 2; Supplementary data).

Data analysis revealed that the mean species richness per sample was not affected by forest age (*F* = 2.3684; *df* = 1, *P* = 0.16) nor by fire (*F* = 0.0947; *df* = 2, *P* = 0.91) (Tables 3). The Shannon index was not affected by forest age (*F* = 0.8327; *df* = 1, *P* = 0.39) nor by fire (*F* = 0.2074; *df* = 2, *P* = 0.82). Similarly, forest age (*F* = 1.1754; *df* = 1, *P* = 0.31) and fire (*F* = 1.9413; *df* = 2, *P* = 0.21) had no effect on evenness (Tables 3).

**Table 3:** Least square mean and SEM for analyzed parameters in control and burned, young and mature Scots pine forests

Parameter	Forest				SEM
	Young		Mature		
	Control	Burned	Control	Burned	
Abundance (m^2^)	2167^bx^	1383^by^	3817^ax^	2150^by^	280.25
Species number	3.33	3.13	4.33	4.13	0.459
Shannon index (*H*′)	0.94	0.98	1.01	1.17	0.150
Evenness	0.863	0.9	0.75	0.85	0.037

Superscripts ^a,b^ indicate statistical differences among rows, superscripts ^x,y^ indicate statistical differences between groups (control vs. burned) within forest age

**Fig. 3:**
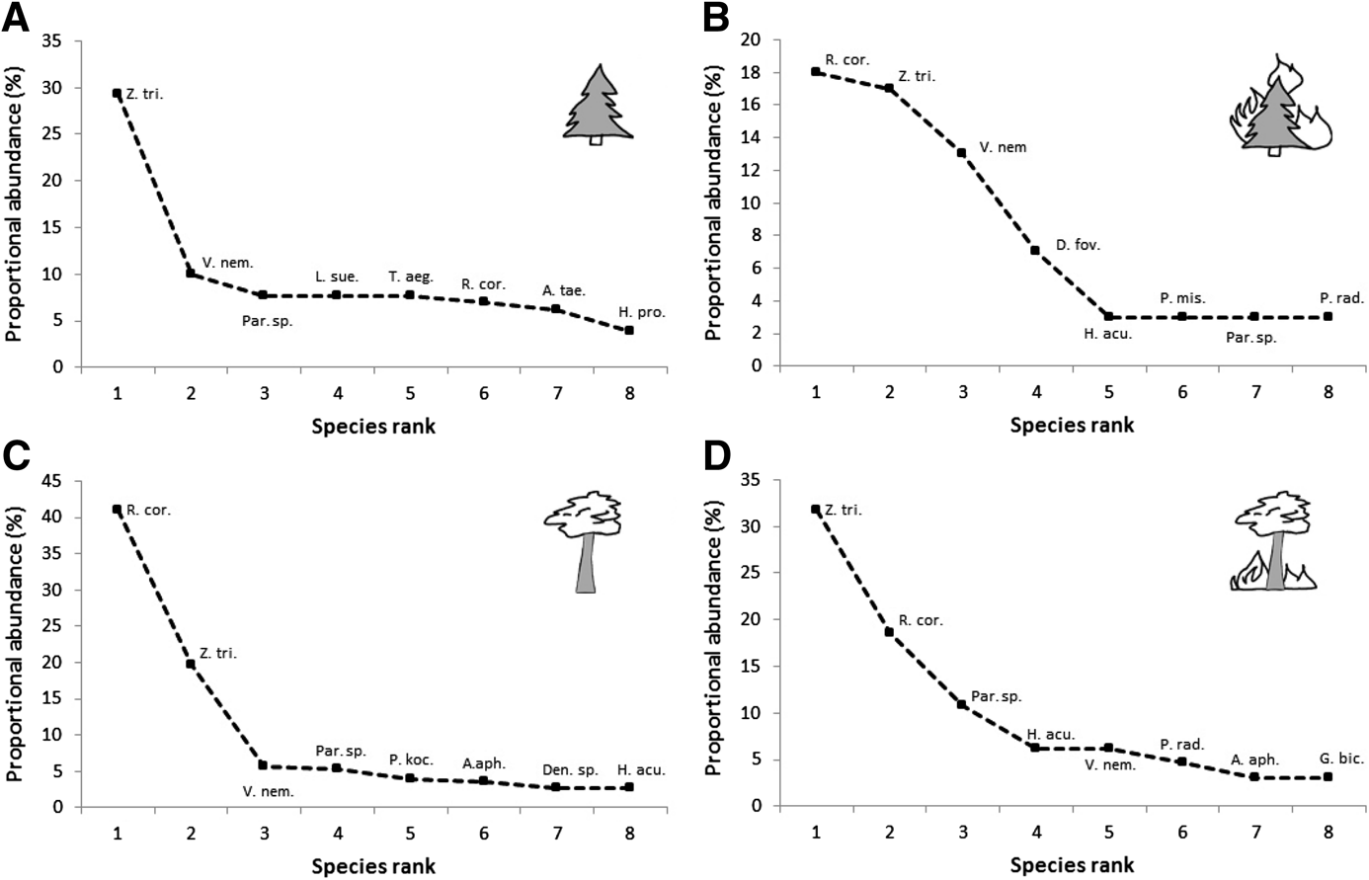
Species rank graphs in young and control (**a**), young and burned (**b**), mature and control (**c**), mature and burned (**d**) forests. See text for full species names

Analysis of the species rank graph revealed changes in the mite community, after the fire, both in young and mature forests, however, changes differed between studied forests (Fig. 3). The mite community in young control plots was dominated by only one species *Zercon triangularis* (29.2% of the total abundance) and the proportional abundance of the other species (*Veigaia nemorensis*, *Paragamasus* sp., *Leptogamasus suecicus*, *Rhodacarus coronatus*, *Trachytes aegrota*) was similar and ranges from 10.0 to 6.9%. Moreover, the ratio between the two most abundant species, i.e., *Zercon triangularis* and *Rhodacarus coronatus*, was 4:1 (29.2 vs. 6.9%) on control plots which was changed by wildfire to 1:1 (20.5 vs. 21.7%) on burning plots in young forests. In the mature forests the mite community was generally dominated by the same species; however, the ratio between *Z. triangularis* and *R. coronatus* was inverted. In the control plots, the ratio was 1:2 (19.7 vs. 41.1%) and in burned plots 1.5:1 (31.8 vs. 18.6%).

**Fig. 4:**
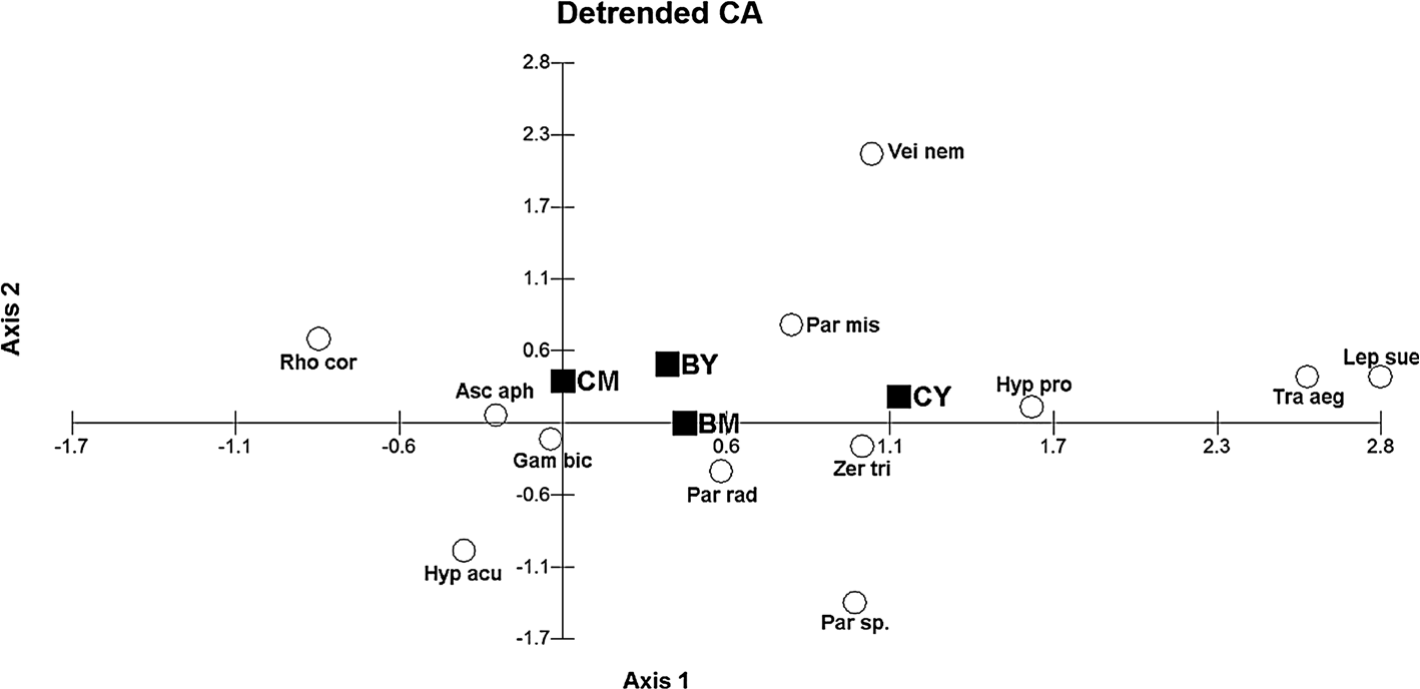
DCA biplot species data for the different microhabitat of the forest floor. Microhabitat are marked as: *BM* burned mature, *BY* burned young, *CM* control mature, *CY* control young. Species abbreviation are as follows: Asc aph—*Asca aphidioides*, Gam bic—*Gamasellodes bicolor*, Hyp acu—*Hypoaspis aculeifer*, Hyp pro—*Hypoaspis procera*, Par mis—*Paragamasus misellus*, Par sp.—*Paragamasus* sp., Lep sue—*Leptogamasus suecicus*, Par rad—*Parazercon radiatus*, Rho cor—*Rhodacarus coronatus*, Tra aeg—*Trachytes aegrota*, Vei nem—*Veigaia nemorensis* and Zer tri—*Zercon triangularis*

Detrended Correspondence analysis (DCA) was performed to evaluate relationships between species abundance and sampled forests (Fig. 4). The eigenvalue was neither significant for axis 1 (*λ*_1_ = 0.223) nor for axis 2 (*λ*_2_ = 0.017); however, over 74.1% of the variance was explained by the first 2 axes and the microhabitats were well separated. Ordination axes are considered as significant when their eigenvalue is higher than 0.3 (Dekkers et al. 1994). The axis 1 ranked the microhabitats from control mature (CM), through burned young (BY) and mature (BM) away from the control young (CY).

## Discussion

In the present study we investigated the influence of two parameters: surface fire and the age of the stand on the abundance and species richness of Mesostigmata mites. Our research revealed that mite abundance is determined by both those parameters – forest age and fire; whereas, the number of reported species is determined only by the age of the stand and the fire does not affect it.

Our study is consistent with other observations in terms of effect of the age on species richness, but contrary in terms of its impact on mite density. Migge et al. (1998) indicated that average density did not differ among forests in various age classes; however, species diversity of oribatid mites tended to be higher in old forests. We have noticed that abundance and species richness are significantly higher in mature forests. This result is in line with Johansson et al. (2016) who studied rove beetles (Staphylinidae) in pine and spruce forests. They proofed that both species richness and abundance increased with forests age, although studies were conducted only in young and middle age stands.

Analysis of the impact of the surface wildfire has confirmed negative influence of the fire only on mite abundance. We have observed decrease of species richness on burned plots, although it was not statistically different compared to control groups. Reduction of mite density due to fire is in line with previous studies on mites (Bogorodskaya et al. 2010), collembolas (Bogorodskaya et al. 2010; Malmström 2012) and other groups of invertebrates (Hylander 2011). Surprisingly, we have not observed a negative effect on species richness. This result can be explained by the nature of surface wildfire analyzed in our study. This type of fire is characterized by low severity, short time of burning and its impact on ecosystem is relatively low (Jung et al. 2010).

We assumed that the surface fire could cause changes in abundance of only those species that occur in the upper layers of the litter and that the species living deeper in the soil would not be threatened by the surface wildfire. Interestingly, the surface fire did not change the proportional abundance of large predators. Furthermore, our studies revealed differences between young and old forest stands. In young stands proportional abundance of large predators such as *Veigaia nemorensis* was similar in burned (15.6%) and unburned (10.0%) plots. In mature forests proportional abundance of this species was similar and ranged on burned and unburned plots 6.2 and 5.7%, respectively. This result can be caused by the buffering of the heat by soil, as was reported by Jung et al. (2010). In that study the temperature decreases from 150 to 300 °C at 10 cm above the forest floor into only 28 °C at 2 cm below the ground.

We assumed that surface fire will affect soil mite community in similar way in both type of forest stands (young and mature). Our hypothesis was not confirmed because the fire affected mite community differently in spite of their similar species composition. In younger forests the proportional abundance of all core species was similar, but the dominant species changed. In young stands *Zercon triangularis* was replaced by *Rhodacarus coronatus* after fire. However, in mature stands the opposite was true, and the fire caused an increase of *Zercon triangularis*. It should be highlighted that in all cases clear domination of one species was observed (Fig. 3).

A clear dominance of *Zercon triangularis* in the controlled young stands can be explained by a common presence of this species in the young pine forest (Kaczmarek 2000). In addition, a higher abundance of *Rhodacarus coronatus* after a surface wildfire may indicate changes in the soil environment and the occurrence of the initial stages of the succession in soil environment. *Rhodacarus coronatus* is one of the r-strategists, which plays a key role in the succession of primary Gamasida on former wasteland (Madej 2004). Moreover, it was previously recognized with numerous annual and perennial crops in the agricultural landscape (Kaczmarek and Ratyńska 1998a, b). This change of dominant species is not astonishing, thus fire causes loss of above-ground vegetation, destruction of litter layer, and release of nutrients (Webb 1994) and also reduction of abundance of oribatids and collembollans (Kim and Jung 2008) which are the food source for some Gamasida mites.

In conclusion, our study revealed that surface fires in Scots pine stands changed the mite community, but it did not change species richness. Soil after surface wildfire creates a favorable environment for species characterized for early succession stages; however, proportional abundance of large predators will not change. Furthermore, our study indicated that the changes in mesostigmatid mite communities depends also on forest age.

## Electronic supplementary material

Below is the link to the electronic supplementary material.

Supplementary material 1 (DOCX 17 kb)
