# Supplementary material for: Mite communities (Acari: Mesostigmata) in young and mature coniferous forests after surface wildfire
Source: Exp Appl Acarol. 2017 Jun 20;72(2):145–60. doi: 10.1007/s10493-017-0148-4 (PMC5486842; doi:10.1007/s10493-017-0148-4)
Supplement: Supplementary file 1 — Supplementary material 1 (DOCX 17 kb) [file 10493_2017_148_MOESM1_ESM.docx]

**Article title:** Mites (Acari: Mesostigmata) communities in young and mature coniferous forests after surface wildfire

**Journal name**: Experimental and Applied Acarology

**Author names:** Jacek Kamczyc^1^, Cezary Urbanowski^1^, Emilia Pers-Kamczyc^2^

**Affiliation:**

^1^Department of Game Management and Forest Protection, Poznań University of Life Sciences, Wojska Polskiego 71C, 60-625 Poznań, Poland

^2^ Institute of Dendrology, Polish Academy of Science, Parkowa 5, Kórnik, Poland

**Corresponding author:** Jacek Kamczyc, jkam@up.poznan.pl, phone 48 061 848 78 03 fax. +48 061 848 78 62

Table 1. Checklist of mite species recorded from control and burned plots in young and mature coniferous forests in Puszcza Knyszyńska Forests

| No. | Species | Control Plots | | Burning Plots | | Total |
| --- | --- | --- | --- | --- | --- | --- |
|  |  | Young | Mature | Young | Mature |  |
| Ascidae | | | | | | |
| 1. | *Arctoseius eremitus* (Berlese, 1918) | 2 | - | - | - | 2 |
| 2. | *Arctoseius semiscissus* (Berlese, 1892) | - | 5 | - | 2 | 7 |
| 3. | *Arctoseius taeniolatus* (Athias-Henriot, 1961) | 8 | - | 1 | - | 9 |
| 4. | *Asca aphidioides* (Linnaeus, 1758) | 2 | 8 | 2 | 4 | 16 |
| 5. | *Asca bicornis* (Can. et Fanz., 188**7)** | - | - | 1 | - | 1 |
| 6. | *Gamasellodes bicolor* (Berlese, 1918) | 1 | 3 | 2 | 4 | 10 |
| Digamasellidae | | | | | | |
| 7. | *Dendrolaelaps cornutus* (Kramer, 1886) | - | - | 1 | - | 1 |
| 8. | *Dendrolaelaps foveolatus* (Leitner, 1949) | - | - | 7 | - | 7 |
| 9. | *Dendrolaelaps* sp. Halbert, 1915 | - | 6 | - | - | 6 |
| Eviphididae | | | | | | |
| 10. | *Alliphis siculus* (Oudemans, 1905) | - | 4 | - | - | 4 |
| 11. | *Eviphis ostrinus* (C. L. Koch, 1836) | 1 | - | - | 1 | 2 |
| Laelapidae | | | | | | |
| 12. | *Hypoaspis aculeifer* (Canestrini, 1883) | 1 | 6 | 3 | 8 | 18 |
| 13. | *Hypoaspis praesternalis* Willmann, 1949 | - | - | - | 1 | 1 |
| 14. | *Hypoaspis procera* Karg, 1965 | 5 | 2 | 1 | 3 | 11 |
| 15. | *Hypoaspis vacua* (Michael, 1891) | 4 | 1 | - | - | 5 |
| Pachylaelapidae | | | | | | |
| 16. | *Pachylaelaps longisetis* Halbert, 1915 | - | - |  |  | 1 |
| Parasitidae | | | | | | |
| 18. | *Holoparasitus calcaratus* (C. L. Koch, 1839) | - | 1 | - | 1 | 2 |
| 17. | *Leptogamasus belligerens* Witaliński, 1973 | - | 3 | 1 | 1 | 5 |
| 19. | *Paragamasus lapponicus* (Trägarth, 1910) | 1 | 1 | - | 3 | 5 |
| 20. | *Paragamasus misellus* (Berlese, 1903) | 4 | 6 | 3 | 4 | 17 |
| 21. | *Paragamasus* sp. Hull, 1918 | 10 | 12 | 3 | 14 | 39 |
| 22. | *Paragamasus suecicus* (Trägardh, 1936) | 10 | - | - | - | 10 |
| 23. | *Pergamasus crassipes* (Linnaeus, 1758) | 1 | - | 1 | - | 2 |
| 24. | *Pergamasus* sp. Berlese, 1903 | 5 | - | 3 | - | 8 |
| 25. | *Vulgarogamasus kraepelini* (Berlese, 1904) | - | - | 3 | 2 | 5 |
| Phytoseiideae | | | | | | |
| 26. | *Amblyseius* sp. Berlese, 1904 | 1 | 2 | - | 1 | 4 |
| Polyaspidae | | | | | | |
| 27. | *Polyaspinus cylindricus* Berlese, 1916 | 2 | - | - | - | 2 |
| Rhodacaridae | | | | | | |
| 28. | *Rhodacarus coronatus* Berlese, 1921 | 9 | 94 | 18 | 24 | 145 |
| Trachytidae | | | | | | |
| 29. | *Trachytes aegrota* (C. L. Koch, 1841) | 10 | 1 | - | - | 11 |
| Veigaiaiidae | | | | | | |
| 30. | *Veigaia exigua* (Berlese, 1916) | - | 6 | - | - | 6 |
| 31. | *Veigaia kochi* (Trägarth, 1901) | 1 | - | - | - | 1 |
| 32. | *Veigaia nemorensis* (C. L. Koch, 1839) | 13 | 13 | 13 | 8 | 47 |
| 33. | *Veigaia planicola* (Berlese, 1892) | - | 1 | - | - | 1 |
| Zerconidae | | | | | | |
| 34. | *Parazercon radiatus* (Berlese, 1914) | 1 | - | 3 | 6 | 10 |
| 35. | *Prozercon kochi* Sellnick, 1943 | - | 9 | - | - | 9 |
| 36. | *Zercon triangularis* C. L. Koch, 1836 | 38 | 45 | 17 | 41 | 141 |
|  | Total | 130 | 229 | 83 | 129 | 571 |
